# Autophagy Augmentation to Alleviate Immune Response Dysfunction, and Resolve Respiratory and COVID-19 Exacerbations

**DOI:** 10.3390/cells9091952

**Published:** 2020-08-24

**Authors:** Garrett Pehote, Neeraj Vij

**Affiliations:** 1 Michigan State University College of Osteopathic Medicine, East Lansing, MI 48823, USA; pehotega@msu.edu; 2 Department of Pediatrics and Pulmonary Medicine, the Johns Hopkins University School of Medicine, Baltimore, MD 21287, USA; 3 PRECISION THERANOSTICS INC, Baltimore, MD 21202, USA; 4 VIJ BIOTECH, Baltimore, MD 21202, USA

**Keywords:** autophagy, exacerbations, COPD, CF, ALI, ARDS, IPF, COVID-19, CFTR, SARS-CoV2

## Abstract

The preservation of cellular homeostasis requires the synthesis of new proteins (proteostasis) and organelles, and the effective removal of misfolded or impaired proteins and cellular debris. This cellular homeostasis involves two key proteostasis mechanisms, the ubiquitin proteasome system and the autophagy–lysosome pathway. These catabolic pathways have been known to be involved in respiratory exacerbations and the pathogenesis of various lung diseases, such as chronic obstructive pulmonary disease (COPD), cystic fibrosis (CF), idiopathic pulmonary fibrosis (IPF), acute lung injury (ALI), acute respiratory distress syndrome (ARDS), and coronavirus disease-2019 (COVID-19). Briefly, proteostasis and autophagy processes are known to decline over time with age, cigarette or biomass smoke exposure, and/or influenced by underlying genetic factors, resulting in the accumulation of misfolded proteins and cellular debris, elevating apoptosis and cellular senescence, and initiating the pathogenesis of acute or chronic lung disease. Moreover, autophagic dysfunction results in an impaired microbial clearance, post-bacterial and/or viral infection(s) which contribute to the initiation of acute and recurrent respiratory exacerbations as well as the progression of chronic obstructive and restrictive lung diseases. In addition, the autophagic dysfunction-mediated cystic fibrosis transmembrane conductance regulator (CFTR) immune response impairment further exacerbates the lung disease. Recent studies demonstrate the therapeutic potential of novel autophagy augmentation strategies, in alleviating the pathogenesis of chronic obstructive or restrictive lung diseases and exacerbations such as those commonly seen in COPD, CF, ALI/ARDS and COVID-19.

## 1. Introduction

Chronic obstructive and restrictive lung diseases are among the leading causes of mortality worldwide, where acute or recurrent episodes of respiratory exacerbations are not only responsible for significant health care costs and a poor quality of life, but also an increased risk of death [1,2,3,4]. The airway mucosa is a primary route of entry for pathogenic microorganisms (bacteria, virus and/or fungi) and represents an important barrier in preventing the entry of these infectious organisms. In the event that the pathogen evades this mucociliary defense mechanism, the airway epithelial cells utilize complex pathogen clearance mechanisms to restrict the life-threatening exacerbations, where these cells work in collaboration with our immune system, involving both innate and adaptive responses, to launch a robust immune response against the invading pathogens, which if successful, results in pathogen elimination or clearance. Thus, providing proof-of-concept evidence in support of autophagy augmentation strategies for alleviating respiratory exacerbations in chronic obstructive pulmonary disease (COPD), cystic fibrosis (CF), acute lung injury (ALI), acute respiratory distress syndrome (ARDS), and coronavirus disease-2019 (COVID-19).

A plethora of studies have identified the “autophagy-lysosome pathway”, a major cellular degradation system, as one of the important mechanisms involved in regulating the immune-mediated pathogen clearance mechanisms in several infection models [5,6,7]. Evidence suggests that a specific form of autophagy, called “xenophagy”, is responsible for the elimination of bacterial, viral or fungal pathogens [7,8,9,10]. In general, autophagy (mainly macroautophagy) is one of the two components of the cellular homeostatic machinery called the “proteostasis network (PN)” [11,12,13,14], which could be termed as the “master regulator of cellular well-being”, as it regulates all the processes involved in protein turnover in the cell. The other component of the PN is the ubiquitin-proteasome system (UPS), which deals primarily with the degradation of cellular proteins [15,16,17,18,19] which allows protein turnover and a replacement of misfolded proteins, as well as the regulation of vital regulatory proteins involved in a variety of cellular homeostatic processes. In contrast, the autophagy-lysosome pathway can handle the degradation of much broader and larger cargo, such as protein aggregates, lipids, damaged organelles, or infectious agents such as bacteria and viruses [9,20,21,22,23,24]. These two components of the PN play a vital role in maintaining cellular homeostasis by facilitating the removal of these dysfunctional cellular components while maintaining or replenishing the levels of proteins, lipids, etc., via synthetic machinery. Other proteostatic mechanisms include the unfolded protein response (UPR), small ubiquitin-like modifier (SUMO), and the endoplasmic reticulum (ER)-associated degradation (ERAD) pathways. Both SUMO and ERAD have a role in the trafficking of the misfolded cystic fibrosis transmembrane conductance regulator (CFTR), which is the dysfunctional protein responsible for the pathogenesis of CF [25,26]. Furthermore, the dysregulation of UPR has been shown to lead to an exaggerated inflammatory response that plays a role in CF pathogenesis progression and exacerbations [27]. Therefore, it is evident that any dysregulation of the PN components leads to severe life-threatening diseases that includes proteinopathies, neurodegenerative diseases, age-related disorders, and chronic respiratory diseases such as CF and COPD [11,13,15,28,29,30]. 

Respiratory infections serve as a trigger for disease exacerbation, while exposure to tobacco, biomass smoke, and aging, are the leading causes for COPD-emphysema development and progression [4,31,32,33]. We and others have demonstrated that autophagy impairment is the key central mechanism for tobacco, biomass smoke or e-cigarette vapor (eCV) exposure and the age-related induction of inflammatory-oxidative stress, alveolar apoptosis and senescence, leading to COPD-emphysema pathogenesis and progression [33,34,35,36,37,38,39]. Additionally, the above-described causative mechanisms also hamper the pathogen clearance, and thus make the individual more prone to acute or recurrent respiratory infections or exacerbations [35,39,40]. Mechanistically, cigarette smoke (CS)-induced phagocytosis defects involves autophagy impairment, which blunts the clearance and promotes the survival of disease-causing pathogens, thereby facilitating chronic infections or recurrent exacerbations [34,35,36]. 

In addition, we recently demonstrated that exposure to CS and other noxious environmental agents promotes accelerated lung aging, which is another important factor contributing to increased infections in chronic lung disease subjects [33]. In fact, aging per se is responsible for an increased risk of pulmonary infections due to the decline of immunity. Notably, the PN and autophagy are known to decrease over time with age, and therefore, serve a crucial role in determining the increased risk of lung infections in the elderly, by virtue of hampered pathogen clearance mechanisms [13,41]. Therefore, several studies, including our recent reports, demonstrate the therapeutic potential of novel autophagy augmentation strategies for controlling COPD-emphysema pathogenesis and progression [22,42,43].

Autophagy plays a crucial role in the elimination of pathogens, and the pharmacological disruption of autophagy has been shown to impair host defense against *Pseudomonas aeruginosa* (*P. aeruginosa*), while the induction of autophagy facilitates *P. aeruginosa* clearance from murine lungs [5,44,45].

Additionally, autophagy-related 7 (ATG7)-dependent autophagy is reported to have a significant role in murine host resistance to *Klebsiella pneumoniae*, an important respiratory track pathogen [46]. Similarly, during a pulmonary infection with *Chlamydia pneumoniae* in mice, autophagy restricts inflammasome activation, while mice deficient in autophagy demonstrate an increased mortality, highlighting the protective role of autophagy [47]. Immunity against viral infections in the airways is also regulated by autophagy. For example, autophagy deficiency promotes interleukin (IL)-17-mediated lung pathology in mice infected with respiratory syncytial virus (RSV) [48]. Furthermore, a recent study demonstrates that IL-22 inhibits RSV production by blocking the virus-mediated suppression of cellular autophagy [49]. Moreover, recent studies on Middle East respiratory syndrome (MERS) coronavirus [50] provide proof-of-concept evidence on the therapeutic potential of autophagy modulating drugs for combating the SARS-COV2 infection, cytokine storm, and pathogenesis of severe ARDS-like COVID-19 fatal lung disease.

Another elaborately studied example where autophagy regulates inflammatory-oxidative stress, inflammation, and infection-mediated disease exacerbations is CF. CF is a genetic disorder, wherein the ΔF508-CFTR is the most common mutation leading to lack of membrane-resident functional CFTR, where the absence of the functional CFTR on the plasma membrane (PM) deteriorates the pathogen clearance ability in CF subjects, leading to persistent infections and chronic inflammation, culminating into a catastrophic lung function decline [51,52,53]. Studies in CF cell lines and knockout mice suggest that the absence of CFTR by itself is sufficient to promote a pro-inflammatory milieu, at least in part, by the activation of nuclear factor kappa-light-chain-enhancer of activated B cells (NFκB) signaling [54,55,56,57]. Moreover, a transient transfection of wild type (WT)-CFTR reduced tumor necrosis factor α (TNFα)-mediated NFκB activation, confirming the anti-inflammatory role of CFTR [55]. Furthermore, a *P. aeruginosa* infection in CF mice leads to a more pronounced NFκB-mediated inflammatory response and pathogenesis of CF-related lung disease [51,58]. Thus, membrane-resident functional CFTR is demonstrated to be a critical regulator of innate and adaptive immune responses, in addition to its classical role as an ion transporter. 

A recent comprehensive article summarizes the role of dysfunctional CFTR in the controlling cellular signaling pathways used by innate immune cells for combating infections such as airway epithelial cells, neutrophils, monocytes, and macrophages [59]. Additionally, we and others have shown a clear role of membrane-CFTR in regulating the function of adaptive immune cells, such as CD3+ T cells, CD4+ T cells, CD4+FoxP3+ regulatory T cells (T regs), and B cells [59]. A recent intriguing study found that CFTR dysfunction in platelets leads to aberrant transient receptor potential cation channel subfamily C member 6 (TRPC6)-dependent platelet activation, which was proposed as a major driver of CF-lung inflammation and impaired bacterial clearance [60]. 

Thus, autophagy plays a vital role in limiting lung infections, and it is evident that a complete or partial absence of functional CFTR leads to autophagy impairment. Mechanistically, CFTR loss or dysfunction results in the reactive oxygen species (ROS)-mediated activation of transglutaminase-2 (TGM-2), and inactivation of the Beclin-1 complex, thereby causing autophagy impairment [61]. Apart from the genetic loss or dysfunction of CFTR, exposure to CS also leads to decreased CFTR activity and expression in vitro, in animal models and in smokers with or without COPD, primarily via ROS-dependent mechanisms [22,42,62,63]. This acquired CFTR dysfunction results in increased inflammatory-oxidative stress, apoptosis, cellular senescence, defective autophagy, and impaired mucociliary clearance, all hallmarks of COPD. 

To summarize, we and others have demonstrated that autophagy augmentation has the potential to not only control CFTR dysfunction-mediated pathologies in CF and COPD, but also allows the clearance of opportunistic infections while balancing immune regulation to avoid recurrent exacerbations and disease progression. This provides a potential to tailor autophagy augmentation for acute and respiratory exacerbations in CF, COPD, ALI/ARDS and COVID-19, etc. Hence, we focus here on the role of autophagy in pulmonary infections and immune dysfunction, where the therapeutic potential of novel autophagy augmenting strategies to alleviate lung disease pathogenesis and respiratory exacerbations is discussed.

## 2. Autophagy, Respiratory Infection and Immunity

Autophagy is the proteostatic process that helps maintain healthy cellular homeostasis by degrading dysfunctional cellular components, lipids, misfolded proteins, etc. [64]. In addition to processing dysfunctional cellular components, autophagy also plays a critical role in immunity and the clearance of pathogens [64]. When working at optimal levels, the autophagy pathway can clear cells of infections via xenophagy, participate in innate and adaptive immune responses, activate macrophages, and remove damaged mitochondria via mitophagy [8,9,20,61,64,65,66,67]. In the respiratory tract specifically, autophagy has even been shown to dictate the length of cilia in the mucociliary escalator that is responsible for the mechanical clearing of pathogens from the airways [68,69,70]. However, the dysfunction and inhibition of autophagy can lead to the pathogenesis of various illnesses.

When it comes to respiratory bacterial infections, autophagy is necessary for a complete immune response. *Mycobacterium tuberculosis* (*M. tuberculosis*) is an intercellular pathogen that causes tuberculosis. *M. tuberculosis* evades the body’s immune response by forming granulomas and blocks phagolysosome trafficking to inhibit the intracellular killing of pathogens [71,72,73]. Recently, studies have demonstrated the importance of functional autophagy in targeting and killing *M. tuberculosis* in respiratory cells by showing that the induction of autophagy pathways overcomes the inhibition of phagolysosome formation to effectively kill *M. tuberculosis* [6,74,75].

In the case of *Streptococcus pneumoniae (S. pneumoniae)*, which is the most common pathogen causing community acquired pneumonia [76], studies have demonstrated that infection activates the autophagy pathway as part of the body’s immune response [77,78]. Recently, it has been shown that this induction of autophagy makes the clearance of *S. pneumoniae* infection more effective by enhancing the rate of phagocytosis by neutrophils [78], demonstrating that autophagy is necessary for an effective immune response in pneumonia. Studies have also exhibited the activation of autophagy in alveolar macrophages by *Pseudomonas aeruginosa* (*P. aeruginosa*) [5,79] and *Klebsiella pneumoniae* [46,79] to induce the degradation of these pathogens in phagolysosomes. Moreover, there has been evidence that autophagy is activated by lipopolysaccharide (LPS), which is a bacterial toxin on the outer membrane of Gram-negative bacteria. Typically, LPS can cause inflammation that leads to lung injury [80,81]; however, the activation of autophagy in response to LPS was shown to attenuate lung injury [82,83]. 

Autophagy is not only integral to the immune response to bacterial infections within the respiratory system, but it has been demonstrated to play an important role in the response to viral pathogens as well [8,9,20,65,84,85]. In the case of the influenza virus, studies have demonstrated autophagy is similarly induced to attenuate the virus and limit its replication [84,85,86,87]. Autophagy has also been shown to be essential in the activation of dendritic cells to produce cytokines and mount an immune response to respiratory synovial virus (RSV) [88,89], MERS [50] and SARS-CoV2 [90,91,92]. Therefore, autophagy is an essential part of the immune response against bacterial and viral pathogens attempting to invade the respiratory tract. 

## 3. Mechanisms of Autophagy Dysfunction

Despite the importance of autophagy, there are many conditions where this process can become dysfunctional, leading to various pathological states. In the lungs, the major sources of autophagy dysfunction includes first- and second-hand cigarette smoke (CS) exposure and aging [35,62,93]. Studies have demonstrated that CS exposure can decrease the expression of transcription factor-EB (TFEB), a master autophagy regulator [34,35]. This occurs by the dysfunctional processing of TFEB in response to CS, causing the perinuclear localization of TFEB which prevents the activation of autophagy [62]. This CS-induced autophagy impairment of TFEB leads to impaired bacterial clearance [22,33,34,35]. Furthermore, there is evidence that CS exposure increases the accumulation of ubiquitinated proteins and p62 (a marker of autophagy impairment) in aggresome bodies, further impairing autophagy [22,34]. These deleterious effects of CS exposure on autophagy have also been demonstrated to be present in eCV exposure, which similarly impairs autophagy through the accumulation of aggresome bodies [39,94]. Furthermore, increasing the severity of pulmonary dysfunction and autophagy impairment has been observed to statistically correlate directly with increased levels of aggresome bodies [36], demonstrating its potential as a prognostic biomarker. Hence, CS exposure demonstrates a common, but preventable way autophagy can be impaired in individuals. Notably, CS exposure accelerates lung aging, known to be initiated by autophagy decline, which can exacerbate infections [33]. 

In addition to smoking, autophagy can be impaired by infectious agents. Due to the conserved nature of autophagy, many organisms have adapted and evolved mechanisms to impair autophagy in order to infect their host. For example, studies have demonstrated that the influenza virus promotes its own survival by preventing autophagolysosome formation; thus, leading to autophagy impairment via aggresome accumulation [41,85,95]. Influenza has also been found to prevent the autophagy-dependent presentation of viral antigens necessary to mount an immune response [10]. Similarly, investigations have found that severe acute respiratory syndrome coronavirus (SARS-CoV) can inhibit autophagolysosome formation with trans membrane papain-like protease 2 (PLP2-TM) to provoke phagolysosome accumulation causing autophagy impairment [96]. Additionally, the nonstructural protein 6 (NSP6) of coronaviruses has also been observed to restrict autophagosome development to prevent cells from inhibiting coronavirus replication [97].

However, autophagy inhibition by pathogens is not something that is unique to viruses. *Legionella pneumophilia* (*L. pneumophilia*) has been found to delay the progression of infected autophagosomes to lysosomes to allow the bacteria to develop an acid-tolerant state prior to autophagolysosome formation [98,99]. This delay allows *L. pneumophilia* to replicate in the acidic environment of the autophagolysosome [99] and cause infection within the host. This mechanism of autophagy impairment is not unique to *L. pneumophilia* and has been observed as a common mechanism of bacteria to promote their invasion and replication [7,100,101]. *M. tuberculosis* has also been observed to impair phagolysosome formation; meanwhile, other studies have further demonstrated that *M. tuberculosis* has the ability to activate cellular pathways that inhibit autophagy within macrophages to promote intracellular survival [61]. Hence, there are various organisms that can infect humans and cause autophagy dysfunction for their own survival. 

The expression of CFTR has been shown to influence autophagy as discussed above. One mechanism of decreased CFTR expression is due to the genetic defects found in CF, where the most common mutation seen in these patients is ΔF508-CFTR [102,103,104]. Studies have demonstrated that ΔF508-CFTR causes protein misfolding that forms aggregates potentially impairing cellular proteostasis [20,42,53,61]. This dysfunction caused by ΔF508-CFTR includes autophagy dysfunction and decreases pathogen clearance in the airways of patients with CF [53]. Similar findings were observed in macrophages with dysfunctional CFTR [105,106]. 

**Figure 1:**
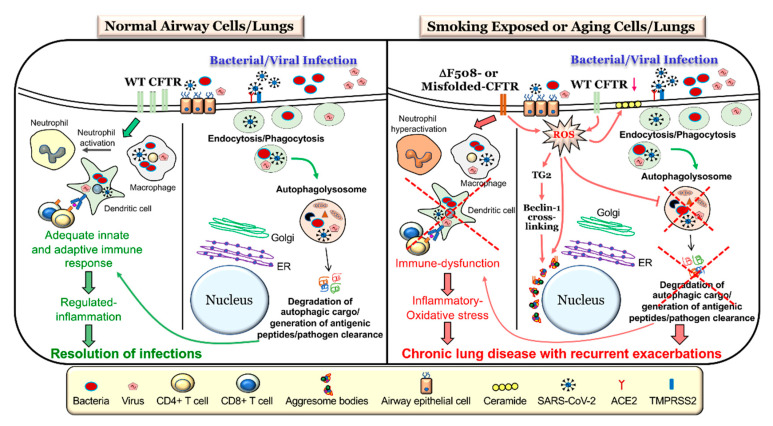
Schematic representation showing mechanisms of respiratory exacerbations and lung disease pathogenesis. The inflammatory/pathogenic receptors and cystic fibrosis transmembrane conductance regulator (CFTR) localized in lipid-raft membranes modulate immune response on viral or bacterial infection of airway cells. In subjects with decreased expression of CFTR (chronic obstructive pulmonary disease (COPD)), misfolded-CFTR (ΔF508 CFTR, cystic fibrosis (CF)), or elderly subjects, increase in reactive oxygen species (ROS) activity within the cells inhibits the progression of endocytosed viruses and phagocytosed bacteria into phagolysosomes. Furthermore, the ROS resulting from misfolded/ΔF508 CFTR or age-related changes causes ceramide accumulation within the plasma membrane, and increases TG2 expression, which causes crosslinking of Beclin-1. This Beclin-1 crosslinking results in perinuclear aggresome body formation that further impairs autophagolysosomes formation to degrade autophagic cargo and clear infectious pathogens. As a result of this impaired degradation or clearance, the immune response is further impaired leading to more ROS formation. This ultimately develops into chronic lung disease with recurrent exacerbations and infections. In case of viral infections such as SARS-CoV-2, the virus binds to the ACE2 receptor TMPRSS2 complex, to fuse with the host cell and gain entry for replication. Autophagosome-lysosomal processing is a standard mechanism for clearance of viruses and other pathogen via xenophagy, which when impaired results in exacerbation, chronic inflammation, and pathogenesis of severe lung disease.

Along with the genetic mechanism of CFTR dysfunction present in CF that can impair autophagy, CFTR function has been shown to be diminished by CS exposure [40,107,108,109], which is known as acquired CFTR dysfunction. One mechanism of CS-induced CFTR dysfunction leading to impaired autophagy is through increased ceramide accumulation as a result of altered sphingolipid homeostasis in COPD patients [22,93,110]. CS exposure can also increase ROS which causes CFTR to accumulate in aggresome bodies and impair autophagy [42,62]. Thus, both genetic mechanisms and CS exposure or environmental factors play a critical role in CFTR dysfunction and the resulting autophagy impairment (Figure 1).

## 4. Autophagy Dysfunction in Respiratory Diseases

A number of recent studies have identified autophagy dysfunction as the central mechanism of elevated inflammatory-oxidative stress, alveolar apoptosis, cellular senescence, and recurrent infections, all of which contribute to the pathogenesis and progression of acute and chronic respiratory diseases [22,35,61,62,70,111]. The inherent ROS in the CS, and the resulting increase in cellular endogenous ROS post-CS exposure, symbiotically contribute to extremely high intracellular ROS levels, which creates an imbalance in the oxidant–antioxidant ratio [22,34,61,112]. This serves as the basic mechanism for lung cellular injury, tissue damage, and the pathogenesis of chronic obstructive or restrictive lung diseases, such as COPD-emphysema (obstructive) and idiopathic pulmonary fibrosis (IPF, restrictive) [37,38,61,113,114,115,116]. Several groups including ours have demonstrated that elevated ROS levels are the key upstream driver of autophagy dysfunction, as treatments with antioxidant drugs rescues the age-related, or smoke, or eCV exposure-induced autophagy defect [15,22,42,61,62].

### 4.1. Autophagy Defects in Acute Lung Injury (ALI)

Extensive studies utilizing both in vitro and in vivo models of acute lung injury have demonstrated the protective role of autophagy via regulating inflammatory-oxidative stress, apoptosis, and pathogen clearance mechanisms, even though there were some initial opposing studies [117,118,119], the subsequent detailed evaluation validates the protective role as discussed. In general, autophagy is induced upon exposure to common triggers of ALI, such as LPS, bacterial infections, hyperoxia, sepsis, etc. [120,121,122]. There seems to be a consensus that autophagy indeed plays a protective role in LPS-induced acute lung injury and inflammation [82,83,123]. In support of this, LPS-mediated severe lung injury in mice, as quantified by lung edema, elevated leukocyte infiltrations, hemorrhages, and increased inflammatory cytokines (IL-1*β* and TNF*α*) in the bronchoalveolar lavage fluid (BALF), was further exacerbated by autophagy inhibition, thereby suggesting its protective role [82]. Mechanistically, the activation of the mammalian target of rapamycin (mTOR) and the resulting autophagy dysfunction has been implicated in promoting LPS-induced lung injury, possibly through the activation of NFκB signaling [124,125]. Further evidence comes from studies demonstrating the utility of autophagy inducers and/or antioxidant drugs in ameliorating LPS-induced acute lung injuries, while treatment with autophagy inhibitors reversed the beneficial effects [125,126]. Autophagy is also protective in sepsis-induced lung injury, as a deficiency of proteins interacting with C-kinase 1 (PICK1) in mice leads to defective autophagy, and more severe acute lung injury in the cecal ligation and puncture (CLP) model of sepsis, as compared to WT animals [120,127]. Additionally, in murine models of hyperoxia-induced ALI, which resembles features of bronchopulmonary dysplasia (BPD), autophagy is proposed as a protective mechanism, and markers of defective autophagy are found in the lungs of human neonates with established BPD [128]. Another recent study describes the suppression of autophagy as a critical mechanism of chronic parenteral nutrition-mediated lung injury, and treatment with the autophagy inducer, rapamycin (an mTOR inhibitor that initiates nucleation, autophagosome elongation, autophagosome maturation, and autophagosome termination), reversed the lung injury features in the animal model of parenteral nutrition [129,130]. Finally, mice deficient in crucial autophagy proteins such as *Atg7*, *Atg5* and *Atg4a* demonstrate more severe ALI features [83,121,130,131], thus confirming the protective role of autophagy in ALI. 

### 4.2. Autophagy Defects in Acute Respiratory Distress Syndrome (ARDS)

More severe forms of ALI may progress to acute respiratory distress syndrome, or ARDS. Autophagy has been shown to play a critical role in regulating the outcome of ARDS [120]. The clinical manifestations of ARDS are very severe and may lead to rapid lung function decline and death. Using one of most widely used murine models of ALI, the CLP, a recent study demonstrated that autophagy induction by rapamycin was able to improve the survival rate, histological scores, lung wet/dry ratios, PaO_2_/FiO_2_, and inflammatory cytokine and myeloperoxidase (MPO) levels in BALF, suggesting a protective role of autophagy in sepsis-induced ALI/ARDS [120]. In another study, the potential of BML-111, a lipoxin A4 receptor antagonist, was evaluated in controlling LPS-induced septic ALI/ARDS in rats. The authors showed that BML-111 inhibited apoptosis and induced autophagy in alveolar macrophages, in response to the LPS challenge, via the suppression of MAPK1 and MAPK8 signaling and was independent of mTOR [132]. Moreover, BML-111 controlled the LPS-induced production of pro-inflammatory cytokines, and reduced apoptosis in the rat lungs, and thus warrants further investigation in ALI/ARDS [132]. Mechanical ventilation (MV)-induced lung injury is another severe form of ARDS, wherein the activation of inflammasome has been shown to mediate ALI symptoms. In a recent article, starvation-induced autophagy augmentation was shown to protect against LPS- and MV-induced ARDS features by reducing IL-1*β* levels, decreasing lung permeability, and improving arterial oxygenation [133]. Thus, autophagy had a protective role in controlling the inflammasome activation and resolution of the MV-induced production of IL-1*β*, which plays a pathogenic role through inducing hypoxemia and increasing lung permeability in LPS/MV-induced ALI/ARDS [133]. Although some contrasting reports regarding the role of autophagy in ALI/ARDS were initially reported [134], there has been a consensus on the therapeutic advantage of autophagy augmentation in ALI/ARDS based on significant validation studies [64,120,122]. Thus, proper autophagy function is essential in attenuating the severity of ARDS in patients.

### 4.3. Critical Role of Autophagy in COVID-19 Exacerbations

The ongoing SARS-CoV-2 pandemic has severely impacted quality of life with a significant health care and socio-economic burden globally. In general, the cellular endocytic and autophagy pathways contribute to viral entry and replication, and thus are obvious attractive targets against SARS-CoV-2 and other viral infections [90,91,92]. The SARS-CoV-2 virus, which causes COVID-19, is highly infectious and can cause cytokine-storm leading to pneumonia and severe lung damage in susceptible subjects by triggering an ARDS-like lung disease, with high-risk of mortality [135]. Recent evidence suggests that SARS-CoV-2 may inhibit autophagy, which we anticipate as a potential mechanism for severe COVID-19 lung disease due to impaired viral clearance and immune dysfunction. In a recent study of the SARS-CoV-2 genome, researchers found that the NSP6 protein of the virus binds with greater affinity to the endoplasmic reticulum (ER) [136]. This genetic change may allow the virus to inhibit autophagy via impaired autophagosome processing, which prevents the degradation of viral particles by the lysosome [136]. There is also evidence that PLP2 is over-expressed in SARS-CoV and MERS-CoV cell lines that also allows the virus to inhibit autophagolysosomal formation and autophagy flux and is likely a method of autophagy inhibition in SARS-CoV-2 [96]. These studies suggest potential mechanism by which SARS-CoV-2 inhibits autophagy to infect or circumvent host cells pathogenic clearance pathways and limit an adequate immune response similar to other coronaviruses that warrants further investigation. 

The elimination of viruses by autophagy (sometimes termed as virophagy) has been well described for a variety of viral infections [8,84,85,137]. Although, the virus has multiple ways of entry into the cell, autophagy augmentation provides strategic advantage in reducing viral load by promoting its clearance [90,137]. As a proof of concept, recent study demonstrated the utility of three different autophagy-inducing drugs, spermidine, MK02206, and niclosamide, in restricting SARS-CoV-2 propagation [138]. Autophagy induction and related upregulation of overall immunity helps combat exacerbations and is suggested as an immunity boosting strategy as a preventive measure against COVID-19 [139]. In addition to boosting immunity, dampening viral load, and allowing SARS-COV2 clearance, autophagy induction may provide strategic advantage in the treatment of COVID-19 and prevention of negative outcomes, which makes it a subject of ongoing studies. In support of this, autophagy inhibiting drugs, such as hydroxychloroquine (HCQ) that help dampen the immune response in rheumatoid arthritis, malaria, and other illnesses but weakens cellular ability for viral clearance by the critical homeostatic process autophagy. Despite early claims that HCQ may provide benefit in treating COVID-19 [90,91,92], it has since been deemed unsafe for use in COVID-19 treatment by the FDA based on randomized double blind placebo control trials [140,141]. In addition, earlier studies on Middle East respiratory syndrome (MERS) coronavirus [50] provide proof-of-concept data on therapeutic potential of autophagy modulating drugs to combat SARS-COV2 infection, cytokine storm, and the pathogenesis of severe ARDS-like COVID-19 fatal lung disease. Prior studies showing a protective role of autophagy induction in other models of ARDS demonstrate the scope of autophagy augmenting strategies in combating SARS-CoV-2 infections (Figure 1) and thus is part of ongoing validation and rapid clinical development studies that may help limit the burden and spread of this novel virus.

As discussed above for other lung diseases, targeting autophagy to prevent the replication of SARS-CoV-2 is not the only potential benefit of autophagy augmentation for the treatment of COVID-19, but it may also allow the fine-tuning of optimal inflammatory responses. As now known, the pathogenesis of SARS-CoV-2-mediated severe COVID-19 involves the activation of numerous pro-inflammatory cytokines as part of the aforementioned cytokine storm causing a hyper-inflammatory state [142]. In addition to the destruction of the lungs and ARDS associated with COVID-19, this inflammatory response can cause damage to the cardiovascular, nervous, renal, hepatic, and gastrointestinal systems with wide ranging immediate and long-term consequences [142]. In support of this, studies have demonstrated the role of autophagy in the inflammatory response within the lungs and other organ systems [61]. As mentioned above, autophagy induction has been demonstrated to attenuate lung inflammation when exposed to a pathogen. Thus, autophagy induction to limit the inflammatory response, in addition to infection, has an immense therapeutic potential as an effective treatment for COVID-19 and decreasing the associated mortality and morbidity.

### 4.4. Autophagy Defects in COPD

We and others have described autophagy dysfunction as a prime causative factor utilizing in vitro and animal models of smoke- (cigarette and waterpipe) or eCV (nicotine)-induced lung injury and COPD-emphysema [33,34,39,94,109]. Moreover, these were validated in human subjects where defective autophagy was verified using human lung tissues from COPD-emphysema subjects, where classical autophagy impairment features, such as aggresome bodies, were associated with the severity and progression of the disease [22,36]. These aggresome bodies are perinuclear accumulations of misfolded or aggregated proteins, which are poly-ubiquitinated and co-localize with p62 and the autophagy protein microtubule-associated protein 1 light-chain-3B(+) (LC3B+) bodies, and are the key indicators of defective autophagy flux [33,36,143]. Additionally, we and others have also noted the increase in aggresome body formation in aged mice lungs that correlated with alveolar airspace enlargement (emphysema phenotype), indicating that age-related decline in autophagy contributes to COPD-emphysema development, similar to CS exposure [33]. Moreover, an increase in emphysema severity (GOLD 0-IV) in smokers with a minimal age difference [33] also correlated with an increase in alveolar senescence, indicating the presence of accelerated lung aging in severe COPD-emphysema subject lungs. We further validate smoke-induced accelerated lung aging using aging markers and in vitro and murine models of COPD-emphysema. In further studies, a clear mechanistic and protective role of TFEB, the master autophagy regulator, was observed in CS-induced lung disease models where other pathogenic features of COPD-emphysema, such as inflammatory-oxidative stress, senescence, apoptosis, and aggresome formation, were used for the validation of pathogenic roles [34,35]. In fact, the CS-induced sequestration of TFEB protein into aggresome bodies leads to its decreased availability, which prevents its function as a transcription factor to positively regulate the autophagy process [34,35]. Moreover, TFEB-mediated autophagy was shown to be protective against oxidative stress and hepatotoxicity induced by ethyl carbamate (a toxicant in CS) [144], suggesting that TFEB-autophagy is a protective mechanism against CS exposure-induced toxicity, not only in the lungs, but in other vital organs as well. Additionally, TFEB-mediated autophagy has also been shown to control CS-induced cellular senescence, and bacterial phagocytic clearance, thus highlighting its protective role in CS-induced COPD-emphysema [22,34,35]. In addition to TFEB, other mechanistic mediators of autophagy have been shown to participate in the sequential dysfunction or impairment of autophagy processes, contributing as a key mediator of COPD-emphysema pathogenesis. For example, increased levels of bicaudal D1 (BICD1), an adaptor for the dynein–dynactin motor complex, were found in the peripheral lung tissues of COPD patients, which was associated with increased p62 oligomers [145]. Additionally, the exposure of bronchial epithelial cells or mice to CS led to increased BICD1 levels, along with defective autophagosome maturation, and an accumulation of BICD1 with p62 and ubiquitin-associated p62-oligomers, thus confirming the mechanistic role of BICD1 in CS-induced autophagy defects [145].

Dysfunctional autophagy has also been associated with defects in specific cell types of the airway. The secretory cells of the airway, such as the club and goblet cells, play an important role in host defense during infection. Autophagy has been recently shown to be required to maintain the function of club cells, independent of CS exposure [146]. Mice deficient in autophagy protein Atg5, demonstrate a diminished expression of the host defense protein secretoglobulin family 1A member 1 (SCGB1A1) and surfactant proteins A1 and D (Sftpa1 and Sftpd), as well as abnormal club cell morphology [146]. Moreover, a diminished SCGB1A1 expression in club cells correlates with evidence of reduced autophagy in lung tissue from COPD former smokers [146]. CS exposure has also been demonstrated to cause the accumulation of damaged mitochondrial via impaired mitophagy, which has been demonstrated to play a role in COPD pathogenesis disease progression [147,148]. Thus, it can be postulated that CS-induced autophagy dysfunction would further deteriorate the structure and function of club cells, resulting in altered or diminished host defense mechanisms in COPD subjects. 

### 4.5. Autophagy Defects in Cystic Fibrosis

Cystic fibrosis is a chronic obstructive lung disease which is marked by recurrent infections, chronic inflammatory-oxidative stress, and mucus overproduction that contributes to severe airway obstruction. Initial seminal studies by Luciani A et al. established the link between defective CFTR and the presence of aggresome bodies, lung inflammation, and ROS-mediated autophagy inhibition [53]. We and others have not only validated that defective CFTR-mediated ROS-TG2 pathway drives the crosslinking of Beclin-1, which results in the accumulation of misfolded ΔF508-CFTR into p62+HDAC6+ aggresome bodies leading to autophagy dysfunction, but we have also demonstrated the key central role of autophagy in regulating CF pathogenesis and exacerbations [42,58,149,150]. These studies describe the specific role of defective autophagy in CF-related chronic infections and resulting inflammatory-oxidative stress. In addition, CF macrophages demonstrate impaired phagocytic activity and thus CF patients are more prone to bacterial infections, such as *P. aeruginosa* and *Burkholderia cenocepacia* (*B. cenocepacia*) [45,51,58,106,151,152,153]. A recent study investigated the precise mechanism of weak autophagic activity in CF macrophages. Using the technique of reduced representation bisulfite sequencing (RRBS) to determine the DNA methylation profile, the authors found that the promoter regions of *Atg12* in CF macrophages are significantly more methylated as compared to the control WT cells, thereby elucidating a novel mechanism for reduced autophagy activity in CF immune cells [154]. 

In a separate study, an increased expression of the microRNA (Mir)c1/Mir17-92 cluster was identified as a potential negative regulator of autophagy in CF macrophages when compared to normal control cells [105]. Furthermore, the in vivo downregulation of Mir17 and Mir20a, partially restored autophagy gene expression and improved the clearance of *B. cenocepacia* [105]. These studies highlight the importance of autophagy as a key protective mechanism in CF exacerbation, and lung disease pathogenesis and progression.

### 4.6. Autophagy Defects in IPF

IPF is a chronic, progressive, and frequently fatal disease associated with aging and dysfunctional autophagy. It is accepted that accelerated epithelial cell senescence plays a vital role in IPF pathogenesis by virtue of atypical epithelial–mesenchymal interactions, and insufficient autophagy is attributed as a mechanism of accelerated epithelial cell senescence and myofibroblast differentiation in IPF [155]. Bleomycin is widely used as a model of drug-induced lung fibrosis. The first study to describe the protective role of autophagy in bleomycin-induced lung fibrosis used the Atg4b-deficient mice model [156]. After 7 days of bleomycin treatment, these mice demonstrated a significantly higher neutrophilic infiltration and inflammatory cytokine production as compared to untreated mice [156]. Additionally, after 28 days of bleomycin treatment, mice developed extensive lung fibrosis, which was accompanied by an elevated collagen deposition and deregulated expression of extracellular matrix genes [156]. Similarly, in mice deficient in LC3B (LC3B^−/−^), bleomycin-mediated lung injury and fibrotic changes were more pronounced, suggesting the protective role of autophagy in bleomycin-induced lung injury and the resulting development of fibrotic lung disease in mice [157]. Another recent study describes the protective role of the anti-inflammatory cytokine IL-37 in the IPF murine model [158]. A further mechanistic delve into the mechanism of IL-37-mediated protection showed that it induces Beclin-1-dependent autophagy while downregulating TGFβ1-mediated lung fibroblast proliferation [158]. Moreover, IL-37 also decreased inflammation and collagen deposition in bleomycin-treated murine lungs while the protective effect was reversed by treatment with 3-methyladinine (3MA), an autophagy inhibitor [158]. Thus, it is plausible that a decrease in IL-37-mediated autophagy might be involved in the progression of IPF. Moreover, the protective effects of autophagy are apparent from this and the other mechanistic studies mentioned above. 

Additional mechanistic evidence comes from a study that showed bleomycin directly binds to annexin A2 (ANXA2) in lung epithelial cells, thereby preventing the nuclear translocation of TFEB; thus, there is an inhibition of the autophagy flux resulting in fibrotic lung disease pathogenesis [159]. Moreover, torin1-mediated TFEB activation restores autophagy flux and ameliorates bleomycin-induced pulmonary fibrosis [159]. Autophagy dysfunction is also reported in human lung fibroblasts from IPF patients, and it is believed that defective autophagy is required to maintain a cell death-resistant phenotype in IPF fibroblasts, suggesting that autophagy dysfunction is a profibrotic mechanism and promotes IPF pathogenesis [64]. Hence, in age-related IPF pathogenesis, autophagy declines with age and the resulting imbalance of inflammatory-oxidative responses is anticipated to mediate the initiation of fibrotic pathophysiology. However, further clinical evaluation is needed to evaluate the therapeutic potential of autophagy augmentation in IPF patients. 

## 5. Autophagy-Mediated CFTR and Immune Response Dysfunction

The genetic loss of CFTR or a decrease in its expression and/or activity due to environmental insults such as CS leads to autophagy dysfunction. An investigation of CFTR-deficient mice or cells isolated from CF subjects revealed an intrinsic defect in autophagy in the absence of CFTR, and the mechanism of defective CFTR-mediated autophagy impairment via the ROS-TG2-Beclin-1 pathway is well established [22,42,53,150]. Supporting studies demonstrate that autophagy augmentation restores CS-induced CFTR dysfunction, inflammatory-oxidative stress, ceramide accumulation, and COPD-emphysema pathogenesis by rescuing aggresome-bound mutant ΔF508-CFTR to the plasma membrane (PM) [22,35]. Conversely, restoring CFTR levels by S-nitrosoglutathione (GSNO) augmentation corrects CS-induced autophagy dysfunction, inflammatory-oxidative stress and COPD-emphysema features [62]. These findings not only highlight the intricate relationship between CFTR and autophagy but also provide a unique therapeutic opportunity to control exacerbations and chronic lung disease progression. Several reports have suggested that the inherently elevated inflammatory-oxidative stress in CF cells, primarily due to activated NFκB signaling, could be dampened by autophagy augmentation [124,160]. Moreover, it’s conceivable that CFTR dysfunction leads to impaired pathogen clearance, as the autophagy-mediated degradation of both intracellular and extracellular pathogens (xenophagy) is well demonstrated [35,45,58,151,161]. An alternate mechanism called LC3-associated phagocytosis (LAP) is also described, which is similar to the normal macroautophagy pathway but does not involve the formation of a double membrane autophagosome [7,162]. Nonetheless, LAP assists in the processing of both intracellular and extracellular pathogens, through the recruitment of LC3 to the phagosomal membrane and subsequent delivery to lysosomes for terminal degradation [7,151,162]. 

Autophagy per se plays a very crucial role in regulating innate and adaptive immunity both in normal conditions and in response to pathogen challenges. Autophagy directly governs key aspects of the innate immune activation, including the secretion of inflammatory mediators, such as cytokines, which are essential to combat different microbial pathogens [7,8,9,84]. The “inflammasome” is a cytoplasmic multiprotein complex that detects pathogenic microorganisms or other cellular stresses and activates potent pro-inflammatory cytokines such as IL-1β and IL-18 [87,133]. Although inflammasome activation is an early innate response to protect the host, its prolonged activation can lead to severe hyperinflammation and tissue injury [47,130]. Recent studies suggest that autophagy is a negative regulator of inflammasome activation as a deficiency of autophagy components such as LC3B or Beclin-1 promotes NLRP3-dependent inflammasome activation [47,130,133]. Apart from its regulation of inflammasome, autophagy also suppresses the overactivation of other inflammation-inducing factors such as NFκB, suggesting a general protective role of autophagy in keeping a check of uncontrolled innate immune activation that may contribute to lung injury [11,37,38,54,55]. In the adaptive immune system, functional autophagy is required for the survival, development, maturation, and function of immune cells such as CD4+ T cells, CD8+ T cells, B cells, neutrophils, and monocytes [7,9,84,163,164,165]. Moreover, in dendritic cells (DC’s), antigen processing and presentation to MHC-II is dependent on the autophagic generation of suitable peptides [9,20,88,163,164,165]. Additionally, antigen presentation to CD8+ T cells on MHC-I is also dependent on autophagy, thus implicating its indispensable role in generating an adequate immune response to bacterial and viral pathogens [163,164,165]. Hence, autophagy promotes adequate antigen presentation to T cells in numerous infection models, thereby facilitating a robust adaptive immune response and further demonstrating the necessary functional role autophagy plays in infections. 

The lack of functional CFTR in macrophages results in an increased production of inflammatory cytokines, and impaired pathogen clearance capacity. The diminished expression of HLA-DQ and HLA-DR (MHC-II molecules) on monocytes derived from ΔF508-CFTR homozygous CF subjects [166], might explain the impaired pathogen clearance ability of CF macrophages. In addition, CFTR deficiency has been implicated in diminished Treg effector function and a more pronounced Th2-biased immune response [167]. The CF defect has also been shown to affect the activation of neutrophils [168], which are the cells responsible for the first line of defense in the airways. Although, airway epithelial cells and lung-resident macrophages sense the invading pathogens and secrete a plethora of factors to induce the recruitment and activation of neutrophils, neutrophils from CF subjects have diminished phagocytic potentials by virtue of the reduced cell surface expression of toll-like receptors (TLRs) and disrupted chloride transport to the phagolysosome [169,170]. Thus the CFTR mutation in CF results in impaired bacterial killing [171]. Moreover, CF neutrophils also demonstrate an increased capacity to release their primary granule contents such as MPO and neutrophil elastase (NE). The uncontrolled release of these enzymes causes lung tissue damage and severe airway inflammation. We have shown that *P. aeruginosa* LPS-induced MPO levels can be reduced by treatment with the histone deacetylase (HDAC) inhibitor, suberoylanilide hydroxamic acid (SAHA), plausibly by restoring the trafficking of ΔF508-CFTR, suggesting that a functional CFTR is required to keep a tab on uncontrolled neutrophil activation [172]. Mechanistically, an absence or dysfunction of CFTR in neutrophils results in the deactivation of the guanosine triphosphate (GTP)-binding protein Rab27a, which causes impaired granule exocytosis [168,172]. Several studies now agree that the pharmacological inhibition of CFTR or the mutant ΔF508-CFTR is sufficient to cause deregulated neutrophil activation via the activation of the NFκB pathway, resulting in hyperinflammation [172].

Therefore, autophagy and CFTR share an intimate relationship in terms of regulating the immune response against infections. Moreover, the rescue of mutant CFTR to the PM corrects the autophagy-mediated immune dysfunction, controlling chronic lung disease pathogenesis (Figure 1).

## 6. Autophagy-CFTR Dysfunction Induces Acute and Chronic Exacerbations

As discussed above, many patients with chronic respiratory diseases such as COPD and CF face acute and chronic exacerbations. These pulmonary exacerbations are characterized by a worsening lung function, significantly below what is normal for their conditions [4,31,173]. These exacerbations are caused by environmental exposures or bacterial and viral infections that result in further deterioration of the patient’s condition [4,31,173,174,175], leading to hospitalization for individuals causing increased morbidity and mortality [4,31,173]. Exacerbations also pose a significant financial burden for patients with chronic respiratory diseases [1,2,176,177]. 

Since pulmonary exacerbations are commonly triggered by infections, the autophagy dysfunction seen in these chronic conditions plays a role in the pathogenesis of this state via the mechanisms discussed above. A common pathogen that colonizes the lungs of individuals with chronic respiratory diseases and can lead to exacerbations is *P. aeruginosa* [4,31,44,58,173,178]. Studies have demonstrated that defects in CFTR impair the ability of cells to clear *P. aeruginosa* infections via autophagy, especially in patients with CF [22,40,53]. Furthermore, it has been shown that the induction of autophagy is able to ameliorate autophagy dysfunction and promote the clearance of *P. aeruginosa* infections [35,45,58]. As previously mentioned, CS exposure can also cause CFTR dysfunction, where a recent investigation demonstrated that CS exposure decreased CFTR expression while a treatment of CS-exposed macrophages with the autophagy inducer fisetin, which works by modulating autophagosome degradation, significantly recovered CFTR expression [35]. This same investigation showed that the CS-induced inhibition of CFTR decreased the clearance of *P. aeruginosa* [35]. This impaired clearance was then alleviated by autophagy induction with fisetin, supporting the findings of other previously mentioned studies [35]. Hence, CFTR-autophagy dysfunction in chronic lung diseases such as CF and COPD leads to *P. aeruginosa* exacerbations. 

Similarly, *Burkholderia cepacia (B. cepacia)*, an opportunistic infection that afflicts 3–5% of CF patients, can provoke exacerbations [151]. It has been found that macrophages can kill *B. cepacia* within autophagolysosomes; however, macrophages with ΔF508-CFTR lacked the bacteria in autophagolysosomes indicating dysfunction [151]. When these macrophages were treated with the autophagy inducer rapamycin, ΔF508-CFTR macrophages were able to fight the *B. cepacia* infection and reduce the resulting inflammation [153,179]. This demonstrates the importance of CFTR-dependent autophagy in not only clearing bacteria that can provoke pulmonary exacerbations, but also decreasing inflammation that can lead to worsened lung functions as well. 

In regard to viral infections, CFTR-autophagy dysfunction limits viral clearance as well. In a recent study, it was determined that CFTR-deficient mice infected with RSV had an impaired ability to clear the virus compare to control mice [180]. Similarly, despite similar cytokine responses, respiratory epithelial cells with ΔF508-CFTR had a higher viral load when infected with rhinovirus in comparison to control cells [181]. From what we know about CFTR-autophagy’s role in pathogen clearance, it is likely that the observed findings in this study are a result of decreased autophagy in the ΔF508-CFTR cells that leads to decreased rhinovirus clearance and a higher viral load. Thus, proper CFTR-dependent autophagy function is necessary for the clearance of both bacterial and viral pathogens in the airway. 

Besides infection, CFTR-autophagy dysfunction can lead to an impaired clearance of cellular debris and products that would normally be degraded. One such product is ceramide, which in response to CFTR-autophagy dysfunction can accumulate in aggresome bodies within cells [22,110,182,183,184,185]. Ceramide accumulation has been observed in both CF patients with CFTR mutations [182,183,186] and COPD patients who have acquired CFTR dysfunction due to CS exposure [22,110].

In the presence of CS, ceramide has been found to accumulate in p62+ aggresome bodies, indicating autophagy impairment [22]. As CFTR-autophagy becomes impaired in both CF and COPD, cells have chronically elevated intracellular levels of ROS altering cellular homeostasis, causing exacerbations and accelerating the pathogenesis and progression of these respiratory diseases [22,110,182]. Moreover, the deleterious effect of ceramide accumulation on CFTR-autophagy has been demonstrated to directly impair defenses against bacteria [52,184,186,187] and viruses [188]. As such, ceramide accumulation as a result of either inherent or acquired CFTR dysfunction leads to autophagy impairment causing a diminished immune response and bacterial colonization or viral replication. Hence, dysfunctional CFTR and autophagy dysfunction go hand in hand with an increased susceptibility of individuals to pathogens that significantly decrease lung function and cause acute and chronic exacerbations (Figure 1).

## 7. CFTR- Autophagy Dysfunction and Pathogenesis of Chronic Obstructive Lung Diseases

The homeostatic autophagy process in the airways is prone to dysregulation by a variety of factors such as exposure to first- or second-hand CS [40], eCV [39], biomass smoke [14,43], waterpipe smoke (WPS) [94], wildfire smoke [189], environmental pollution [61], genetic polymorphisms [53,61,149], aging [190], obesity [191,192], and changes in the expression and/or activity of CFTR [22,35,53]. Elevated levels of ROS/RNS ensuing from exposure to the above-described risk factors is the primary driver of autophagy impairment (Figure 1), which is considered a central mechanism for the advent of inflammatory-oxidative stress, cellular senescence, and apoptosis [62,94]. These potentially deleterious changes result in the initiation and progression of COPD-emphysema, as they correlate with the severity of emphysema in COPD subjects [22,33,62]. We and others have demonstrated the important role of transcription factor-EB (TFEB), the master autophagy regulator, in controlling inflammatory-oxidative and immune signaling [34,143,193]. In a relatively recent study, we showed that CS-induced accumulation or trapping of TFEB proteins into perinuclear aggresome bodies prevents it from entering the nucleus and performing its function as an autophagy regulating transcription factor, thereby culminating in autophagy dysfunction [34]. These findings were confirmed in human lung tissue sections from smoker and nonsmoker COPD subjects, where the perinuclear accumulation of TFEB into aggresome bodies increases with emphysema severity [34]. Moreover, smokers showed a more prominent increase in TFEB-aggresome localization as compared to nonsmokers, emphasizing that CS exposure leads to defective autophagy via functional trapping of TFEB into aggresome bodies [34]. It is evident from innumerable studies that a complete loss or decrease in the levels of functional WT-CFTR from the PM due to genetic mutations leads to several deleterious changes in the airways that ultimately results in obstructive lung disease pathogenesis in CF subjects. Specifically, a decrease in/loss of WT-CFTR is causally related to increased ROS-mediated inflammatory-oxidative stress, mucus hypersecretion, elevated ceramide levels, and hampered mucociliary clearance resulting in an increased incident of recurrent and chronic pulmonary infections, all of which result in chronic obstructive pathologies in CF airways [15,51,53,110,194]. It is now well documented that CS exposure [22,40,63,107,108], eCV [39,195], or certain infectious agents [196,197,198] also induce a decrease in the activity or expression of CFTR in the airways and was aptly described as acquired CFTR dysfunction. As expected, CS-induced ROS and the resulting oxidative stress was found to be the main cause of acquired CFTR dysfunction in COPD [62,107,108,109]. Moreover, the mechanistic confirmation of the role of acquired CFTR dysfunction in COPD pathogenesis comes from studies which showed that the pharmacological rescue of mutant CFTR to the PM was able to correct CS-induced inflammatory-oxidative stress, autophagy impairment, and COPD-emphysema pathogenesis [14,42,62]. Another important mechanism which plays a crucial pathogenic role in both CF and COPD-emphysema is ceramide accumulation (Figure 1), and numerous studies have highlighted that a loss or decrease in CFTR and/or CS exposure leads to an increase in ceramide levels [22,52,93,110,182,183,186,188]. Our earlier studies described a direct correlation between a lack of lipid-raft CFTR expression and CS-induced apoptosis along with defective autophagy and the progression of COPD-emphysema via ceramide or lactosylceramide accumulation [93,110]. Lately, we also demonstrated that autophagy augmentation alleviates CS-induced CFTR dysfunction, ceramide accumulation (lipophagy impairment), and resulting COPD-emphysema pathogenesis [22], thus demonstrating that autophagy and CFTR share an interconnected biology crucial for the initiation and progression of chronic lung diseases (Figure 1). We further went on to demonstrate that CS-induced autophagy dysfunction and the dysfunction of its component lipophagy lead to intracellular ceramide accumulation [110]. Meanwhile, acquired CFTR dysfunctions caused ASM activation-dependent membrane ceramide accumulation [22]. The pathogenic role of ceramide has also been implicated in CF lung disease, wherein it mediates the inflammation, apoptosis, and increased susceptibility to *P. aeruginosa* infection [183,186,199], and is elevated in the airways of CF mice and patients. Mechanistically, it was proven that the ROS-dependent activation of acid sphingomyelinase (ASM) resulted in increased membrane ceramide accumulation [200]. Thus, several ASM inhibitors have been tested and were shown to reduce ceramide accumulation along with resulting infection and inflammation in the lungs of CF mice [186,200]. Therefore, there is strong evidence that CFTR-autophagy dysfunction is a prime factor that promotes multiple host destructive phenomena, including inflammatory-oxidative stress and recurrent infection-related exacerbations, which eventually contribute to chronic obstructive lung disease pathogenesis.

## 8. Autophagy Augmentation Strategies to Mitigate the Pathogenesis and Progression of Chronic Obstructive Lung Diseases

As discussed above, autophagy-CFTR dysfunction plays a vital role in regulating the pathogenesis of chronic obstructive lung diseases, including facilitating recurrent infections leading to severe disease exacerbations and an increased risk of mortality. Therefore, it is apparent that pharmacological interventions targeted to correct the autophagy-CFTR dysfunction provides a lucrative therapeutic strategy to control chronic obstructive lung diseases pathogenesis. Indeed, using in vitro and pre-clinical murine models, we and others have shown that autophagy augmentation mitigates several pathogenic features of chronic lung diseases, such as inflammatory-oxidative stress, apoptosis, cellular senescence, lung tissue damage, and bacterial or viral infections [22,35,42,45,58,62,149,151,153]. The utility of pharmacological or natural compounds that can alleviate autophagy-CFTR dysfunction has been comprehensively investigated in both CS-induced in vitro and pre-clinical murine models of CS exposure, with or without *P. aeruginosa* co-infection [34,35,42]. We have extensively tested the pre-clinical therapeutic efficacy of cysteamine, a naturally occurring FDA-approved aminothiol compound, which is a known proteostasis and autophagy regulator that induces autophagosome formation, in controlling various pathogenic features of CS- and aging-induced inflammatory-oxidative stress, apoptosis, cellular senescence, pathogen clearance, lung injury, and COPD-emphysema [22,33,94]. Even though cysteamine offers several beneficial attributes such as its antioxidant, bactericidal, mucolytic, and, the most promising, CFTR-rescuing potential that corrects the CS-induced acquired CFTR dysfunction, there are some limitations such as the optimization of beneficial dose and airway delivery methods. We and others have devised strategies such as nano/dendrimer-based formulations which can be efficiently delivered through intranasal inhalation [42,45,149]. The more specific targeting of pulmonary tissues using nano/dendrimer-based drugs improves the therapeutic potential while mitigating some system-wide side effects that may be associated with systemic and nontargeted delivery methods [42,45,149,201]. We believe that cysteamine or its nano/dendrimer formulations have a significant potential of controlling COPD-emphysema pathogenic features, including recurrent exacerbations, based on its known pre-clinical efficacy and ongoing clinical evaluations in controlling obstructive lung disease. 

Our relatively recent study using GSNO, an endogenously occurring nitric oxide donor, highlights the biological significance of CS-induced CFTR dysfunction-related autophagy impairment in mediating COPD-emphysema pathogenesis. We showed that an augmentation of GSNO decreases cigarette smoke extract (CSE)-induced ROS activation and autophagy-flux impairment by rescuing the aggresome-bound perinuclear CFTR to the PM [62]. Furthermore, using a preclinical COPD-emphysema murine model, we demonstrated that chronic CS (Ch-CS) induced an increase in inflammatory cytokines in BALF, aggresome formation, CFTR-aggresome localization, oxidative/nitrosative stress, and apoptosis, and the emphysematous changes (alveolar airspace enlargement) were significantly improved by augmenting the airway GSNO levels [62]. Thus, this study provides proof-of-concept evidence that GSNO augmentation could be further tested as a potential strategy to correct CS-induced CFTR-autophagy defects. Apart from cysteamine and GSNO, we also tested the potential of other FDA-approved autophagy-inducing drugs, such as gemfibrozil (GEM), which induces lysosome formation, and fisetin, in controlling CS-induced autophagy dysfunction and hampered pathogen clearance. Our study showed that CS/CSE-induced TFEB/autophagy impairment, inflammatory-oxidative stress, apoptosis, and senescence can be mitigated by treatment with GEM/fisetin via TFEB induction [34]. In a subsequent investigation, we demonstrated that CSE-induced autophagy dysfunction in macrophages is a critical mechanism of phagocytosis defects and the resulting diminished clearance of *P. aeruginosa* [33,35]. The autophagy-inducing antioxidant fisetin was able to restore the CS-induced phagocytosis defect and facilitate *P. aeruginosa* clearance [35], suggesting the potential of autophagy-inducing strategies in controlling exacerbations prevalent in COPD-emphysema subjects. 

The therapeutic potential of autophagy augmenting drugs has been extensively investigated in controlling chronic CF lung disease and associated pulmonary infection-related exacerbations. There have been extensive studies on the use of rapamycin in controlling CF-related lung infections, but its clinical use is hampered due to its potent immunosuppressive property and certain reports of significant lung toxicity [5,6,44,151,153,202]. Lately, the efficacy of the thymic peptide, Thymosin α-1 (Tα1), was demonstrated in correcting the basic defect in CF, which is the restoration of misfolded ΔF508-CFTR to the PM [203]. Tα1 possesses anti-inflammatory properties and is also known to induce autophagy [204], which could be its mechanism of action to rescue ΔF508-CFTR. Nonetheless, future pre-clinical and clinical studies will be essential before it could be any therapeutic benefit in CF-related autophagy dysfunction and exacerbations. The utility of cysteamine, a potent antioxidant drug with autophagy-inducing potential, has been widely tested in CF in vitro, in vivo models, and is currently being investigated in phase 2 human clinical trials [58,205,206,207]. However, a previously completed study of 10 patients with the ΔF508-CFTR mutation demonstrated a significant improvement in CFTR function with cysteamine treatment [208]. Similarly, our recent studies validated cysteamine’s extensive repertoire of protective mechanisms in CF, and demonstrated for the first time that cysteamine was able to control CS-induced lipophagy impairment and the resulting ceramide accumulation in murine lungs [22]. This finding might have implications in controlling both COPD and CF-related infections and exacerbations, knowing the deleterious role of ceramide in promoting pulmonary infections in COPD and CF.

In addition, autophagy dysfunction has been now widely accepted as a pathogenic mechanism in IPF, and thus strategies to augment autophagy are justified as relevant potential interventions in controlling IPF pathogenesis [209]. Indeed, some recent studies have shown that autophagy mitigates IPF pathogenesis by regulating the fibroblast apoptosis and senescence of alveolar epithelial cells [209]. Moreover, a recent report describes the utility of IL-37 in reducing the bleomycin-induced inflammation and collagen deposition in murine lungs by increasing Beclin-1-dependent autophagy [158]. 

**Figure 2:**
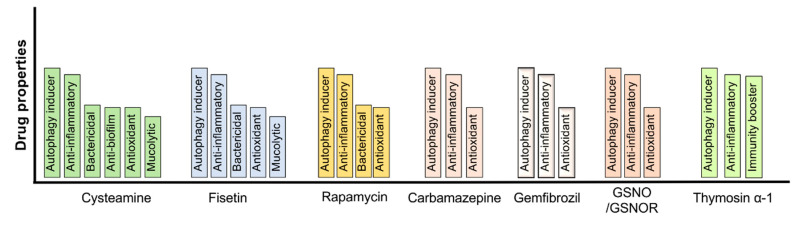
Autophagy-inducing compounds and their potential in treating inflammatory-oxidative stress responses, respiratory exacerbations and coronavirus disease-2019 (COVID-19). The autophagy induction and other properties of various therapeutic compounds for treating pulmonary pathologies and exacerbations are shown. The ability of these compounds to induce autophagy to rescue dysfunctional CFTR or clearance of misfolded proteins from aggresome bodies is a critical therapeutic property to consider when evaluating possible treatments for age-related and respiratory conditions. In addition, anti-inflammatory, antioxidant, anti-biofilm, bactericidal, immune boosting, and mucolytic properties are further aspects of these compounds that have significant therapeutic potential in controlling chronic immune responses and exacerbations that trigger fatal lung conditions such as COPD, CF, acute respiratory distress syndrome (ARDS), idiopathic pulmonary fibrosis (IPF), and COVID-19.

Therefore, autophagy augmentation strategies likely have a strong potential to control chronic obstructive lung disease pathogenesis, including suppressing the severe pulmonary exacerbations which frequently result in patient mortality (Figure 2). 

## 9. Perspective

The burden of exacerbations on the health care system is substantial [1,3,177,210]. For patients with COPD in the United States, the average annual health care costs associated with their condition was estimated to be USD 9981; meanwhile, the average cost to society was USD 30,826 per patient [211]. Similarly, CF exacerbations were demonstrated to cost Medicaid on average between USD 44,589 and USD 116,169 annually with the costs increasing with the age of patients [2]. Another study found that the average cost per episode of all CF exacerbations was USD 12,784 [176]. As such, investigating new methods to prevent recurrent exacerbations is necessary to lower the burden on the health care system while improving the quality of life for patients with chronic pulmonary conditions. The potential of autophagy augmenting therapeutics to correct CFTR-autophagy dysfunction and resulting exacerbations provides a possible solution to this issue (Figure 2). 

As a proof of concept, numerous recent studies have demonstrated the potential of targeting CFTR-autophagy dysfunction as a method for reducing recurrent exacerbations. One drug that has shown promise in alleviating autophagy impairment to provide possible therapeutic benefits in the treatment of exacerbations is cysteamine. Cysteamine is an FDA approved drug for the treatment of cystinosis; however, it also possesses antioxidant, anti-inflammatory, autophagy-inducing, mucolytic, and anti-bacterial properties [58,205,207]. Recently, Ferrari et al. demonstrated that cysteamine could re-establish *P. aeruginosa* clearance in macrophages with the ΔF508-CFTR deletion by salvaging ΔF508-CFTR function in macrophages [58]. This restoration of function allowed macrophages to both increase the internalization and clearance of *P. aeruginosa* via a Beclin-1-mediated initiation of the autophagy pathway [58]. This reveals a possible therapy to restore both CFTR and autophagy function in CF patients that could limit *P. aeruginosa* colonization. An improved clearance of *P. aeruginosa* in CF patients is especially important due to the prevalence of *P. aeruginosa* infections in exacerbations, and a drug that improves the immune response in this way offers a method to decrease exacerbations. 

Studies exploring cysteamine-dendrimers have revealed similar results. Two of our recent studies demonstrated that cysteamine-based dendrimers were able to not only decrease ΔF508-CFTR aggregation, but also rescue the protein to increase the plasma membrane expression [45,149]. Moreover, these investigations showed that this recue of CFTR by the cysteamine-dendrimers alleviated the autophagy impairment associated with the ΔF508-CFTR mutation [45,149]. This alleviation of CFTR-autophagy impairment was further shown to increase the clearance and killing of *P. aeruginosa* in both studies [45,149]. Hence, cysteamine-based dendrimers provide a therapeutic option in the treatment of both acute and chronic exacerbations in CF. Cysteamine’s dendrimer-based formulation also proved beneficial for clearing *P. aeruginosa*, and thus has potential for treating other infections in CF and non-CF patients who are prone to exacerbations. The added advantage of this intervention strategy is that exacerbations in respiratory diseases including those with CFTR dysfunction may be treated and prevented without antibiotics, where multidrug-resistant (MDR) infections are common. 

Further studies into the therapeutic potential of other autophagy inducers for exacerbations in chronic respiratory diseases have shown promise as well (Figure 2). These studies have demonstrated that rapamycin can induce autophagy and aid in the killing of different types of bacteria that are known to cause exacerbations [6,44,153,202]. Similarly, fisetin, which is an over the counter antioxidant used for brain health, induces autophagy [34,43] to improve bacterial clearance by ameliorating CFTR-autophagy dysfunction [35]. Thus, the use of various autophagy inducers shows promise in restoring the CFTR-autophagy pathway to treat exacerbations. However, future studies and clinical trials need to be conducted to evaluate and standardize the safety and efficacy of these treatments to provide benefit in human subjects as a part of ongoing clinical development.

Another reason to explore treatments that restore the CFTR-autophagy function to treat exacerbations is that the current first-line treatment for exacerbations usually includes antibiotics [4,173,210,212,213]. In situations where patients experience severe exacerbations, they are often hospitalized and given powerful intravenous (IV) antibiotics [4,31,173,176]. This is part of what plays into the exorbitant costs associated with recurrent exacerbations. When looking specifically at exacerbations requiring hospitalization and IV-antibiotics, the cost is USD 36,319 per exacerbation for patients [176]. This frequent use of antibiotics in both the out-patient and in-patient treatment of exacerbations causes bacteria to develop MDR, which poses a significant issue for patients as the treatment of future infections becomes more difficult [151,214,215]. In fact, many pathogens that cause exacerbations have been found to have MDR [214,215]. As more bacteria demonstrate MDR, more powerful antibiotics are needed to fight off infections, but these powerful antibiotics can have negative side effects. As such, researchers have begun investigating the potential of targeting the autophagy pathway to fight off the MDR bacteria causing exacerbations. 

One recent study explored the potential of cysteamine to improve the clearance and killing of different MDR bacteria by macrophages in CF patients [206]. The researchers demonstrated that there was a significantly increased clearance of *B. cenocepacia*, *B. multivorans*, and *P. aeruginosa* by alveolar macrophages treated with cysteamine [206]. Further exploration revealed a decrease in the markers of autophagy impairment in these cysteamine-treated macrophages, along with an increase in expression of CFTR on the membrane of the macrophages [206]. Thus, it can be concluded that cysteamine has the ability to promote host clearance of MDR pathogens in pulmonary exacerbations via the alleviation of CFTR-autophagy dysfunction. 

A similar finding was found in a study that explored a novel autophagy inducer that works by initiating autophagosome formation, AR-13. The investigators in this study demonstrated that AR-13 on its own was able to significantly reduce the infectious burden of methicillin-resistant *Staphylococcus* aureus and *P. aeruginosa* in both CF and non-CF immune cells [152]. Meanwhile, in the case of *B. cenocepacia*, AR-13 in combination with antibiotics was able to significantly improve the bacterial clearance from infected macrophages [152]. This improved bacterial clearance was attributed to the alleviation of CFTR-autophagy dysfunction [152]. 

Hence, therapeutics that aim to restore CFTR-autophagy function demonstrate promise in fighting MDR infections that cause exacerbations. If clinical trials of these drugs were to show promise, they may decrease the length of hospitalization and recurrence of exacerbations to decrease both the costs and decreased quality of life faced by individuals with chronic respiratory disease. Moreover, the fact that these drugs have been demonstrated to work by inducing autophagy is critical in fighting MDR bacteria. Since the mechanism involves the host immune response and there would be less of a need for antibiotics, there is the possibility of decreased incidence of MDR bacteria development. 

However, bacteria are not the only pathogens responsible for exacerbations. Viruses are also a common cause of exacerbations in patients with chronic respiratory diseases [4,31,173]. However, the treatment of viruses is significantly different than the treatment of bacteria. For most bacteria, there is an antibiotic that can be prescribed to fight the infection. On the other hand, most viruses tend to be treated with supportive care due to the lack of antiviral therapies for many types of viruses. As such, being able to clear viruses via autophagy/Tvirophagy may provide a unique therapeutic benefit in both reducing the severity of viral infections and decreasing the duration of viral infections, resulting in a decrease in exacerbations. 

The focus on targeting autophagy to treat exacerbations in lieu of antiviral medications is limited but is gaining more interest. One recent study has demonstrated the promise of budesonide, a corticosteroid, in the treatment of rhinovirus infections. Rhinovirus causes the common cold and is a common viral cause of exacerbations in CF [32,175], asthma [32,216], and COPD [32,217]. In this study, researchers found that intranasal budesonide decreased the viral load of rhinovirus in patients’ lungs [218]. The investigators determined the antiviral effect of budesonide was due to autophagy induction as autophagy inhibition with chloroquine and bafilomycin A1 both significantly reduced the antiviral effect of budesonide [218]. Similarly, a study performed with dexamethasone, another corticosteroid, demonstrated that it is an effective treatment of rhinovirus through autophagy induction as well [219]. Thus, there is promise in treating and preventing exacerbations caused by rhinovirus using autophagy-inducing drugs. In theory, targeting the induction of autophagy should provide a therapeutic mechanism for the treatment of other viruses that cause exacerbations in patients with chronic respiratory disease due to the role of autophagy in the immune response in many viruses, yet more research is needed.

In addition to showing promise in targeting viruses that cause exacerbations, the induction of autophagy may also aid in fighting novel viruses that can lead to pandemics. For example, the novel coronavirus SARS-CoV2-mediated human respiratory infection, leading to COVID-19, was first reported in late 2019 [137]. By March 2020, the World Health Organization had declared a pandemic in response to the virus. SARS-CoV-2 has caused numerous deaths, due to lack of a potent effective treatment, where antivirals such as remdesevir, favipiravir, etc., although minimally effective in reducing the hospitalization time of severe lung disease subjects, are in short supply. Moreover due to the novel nature of the virus, susceptible population groups and aerosol-based rapid transmission, we see variations in the control of transmission and mortality between different countries, where mitigation measures are dependent on socio-economic, health care disparities and political will [137,220]. As such, there is a need to rapidly deliver on potent treatments in addition to vaccines, and autophagy augmentation with drugs that are FDA-approved for other conditions (Figure 2), provide significant potential for clinical validation and rapid translation. In fact, a recent pre-print study that we mentioned above has investigated the use of spermidine (an autophagy inducer), MK-2206 (an AKT inhibitor), and niclosamide (a Beclin-1 stabilizer) in the treatment of COVID-19, and has been found to demonstrate some therapeutic benefits as an antiviral treatment against the virus [138]. Thus, autophagy induction may provide a beneficial treatment of the virus. However, further investigation into these drugs and other autophagy inducers is needed. Still, the mechanism of autophagy induction may prove crucial as a treatment of COVID-19 over classical antivirals and provide a tool to end the pandemic. This mechanism may also prevent a future epidemic or pandemic by offering possible treatments to novel viruses without vaccines and antivirals that take significant development time for each strain. 

Therefore, targeting and inducing the autophagy pathway is an area of tremendous clinical significance. Autophagy-inducing drugs may be necessary for overcoming the CFTR-autophagy impairment found in many chronic respiratory diseases to prevent exacerbations and improve the morbidity and mortality for patients while cutting health care costs for these individuals. Moreover, autophagy-inducing drugs offer the ability to fight pathogens without causing MDR bacteria and the need to develop strain specific antivirals. As a result, an autophagy induction intervention strategy to handle MDR bacterial infections or novel viral infections provides significant benefits in terms of controlling exacerbations, whereas autophagy induction with drugs currently approved for other conditions allows rapid translation. Moreover, the proposed autophagy augmenting therapeutic strategy also provides a possible treatment in situations where it is not possible to develop an antiviral for a pathogen. Furthermore, autophagy induction also can offer an effective way to treat current recurring MDR infections. Hence, future clinical studies need to expand their scope beyond the horizon of antibiotics and antivirals for the treatment of many respiratory diseases and begin investigating the therapeutic benefits that autophagy augmenting drugs may have in a myriad of clinical situations. In doing so, we can achieve a significant reduction in the overwhelming health care costs and resources needed for respiratory exacerbations while improving the quality of life with a reduction in the mortality of patients.
